# Organ-specific effects on glycolysis by the dioxin-activated aryl hydrocarbon receptor

**DOI:** 10.1371/journal.pone.0243842

**Published:** 2020-12-15

**Authors:** Silvia Diani-Moore, Tiago Marques Pedro, Arleen B. Rifkind

**Affiliations:** Department of Pharmacology and Pharmacology PhD Program, Weill Cornell Medicine, New York, New York, United States of America; University of Nebraska Medical Center, UNITED STATES

## Abstract

Activation of the aryl hydrocarbon receptor (AHR) by the environmental toxin dioxin (2,3,7,8-tetrachlorodibenzo-*p*-dioxin, TCDD) causes diverse toxicities, including thymus atrophy and hepatosteatosis. The mechanisms by which AHR activation by TCDD leads to these toxicities are not fully understood. Here we studied the effects of TCDD on a major energy pathway, glycolysis, using the chick embryo close to hatching, a well-established model for studying dioxin toxicity. We showed that 24 hr of TCDD treatment causes changes in glycolysis in both thymus and liver. In thymus glands, TCDD decreased mRNAs for glycolytic genes and glucose transporters, glycolytic indices and levels of *IL7* mRNA, phosphorylated AKT (pAKT) and HIF1A, stimulators of glycolysis and promoters of survival and proliferation of thymic lymphocytes. In contrast, in liver, TCDD increased mRNA levels for glycolytic genes and glucose transporters, glycolytic endpoints and pAKT levels. Similarly, increases by TCDD in mRNA levels for glycolytic genes and glucose transporters in human primary hepatocytes showed that effects in chick embryo liver pertain also to human cells. Treatment with the glycolytic inhibitor 2-deoxy-d-glucose exacerbated the effects on thymus atrophy by TCDD, supporting a role for decreased glycolysis in thymus atrophy by TCDD, but did not prevent hepatosteatosis. NAD^+^ precursors abolished TCDD effects on glycolytic endpoints in both thymus and liver. In summary, we report here that dioxin disrupts glycolysis mediated energy metabolism in both thymus and liver, and that it does so in opposite ways, decreasing it in the thymus and increasing it in the liver. Further, the findings support NAD^+^ boosting as a strategy against metabolic effects of environmental pollutants such as dioxins.

## Introduction

Binding of the environmental toxin 2,3,7,8-tetrachlorodibenzo-*p*-dioxin (TCDD) to the aryl hydrocarbon receptor (AHR), a ligand activated transcription factor, activates the AHR and leads to diverse toxic effects. These include thymus atrophy, a consequence of TCDD exposure in all species thus far examined, and hepatosteatosis [1–4], a forerunner of hepatocarcinoma [5]. Thymus atrophy also accompanies aging in mammalian species including humans and is thought to be linked to increased risks of cancer and infection in the elderly [6]. Notwithstanding decades of studies, the cellular mechanisms by which AHR activation by TCDD leads to these and other TCDD toxicities are not fully understood.

We previously reported using chick embryos (CE) close to hatching, a well-established model for studying TCDD toxicity [7, 8], that thymus atrophy and hepatosteatosis caused by TCDD *in ovo* are accompanied by decreased nicotinamide adenine dinucleotide (NAD^+^) levels in those organs. The decrease in NAD^+^ was attributable, at least in part, to NAD^+^ consumption by increased poly-ADP-ribosylase (PARP) activity [9], in particular the TCDD-inducible PARP (TiPARP, PARP7, ARTD14). We found that TCDD enhanced PARP activity leading to suppressed activity of sirtuins, (i.e. SIRT1 and SIRT6) [9, 10], energy regulating enzymes that, like PARPs, require NAD^+^ as a substrate for their catalytic activities [11]. Consistent with these findings, we discovered that NAD^+^ boosting with nicotinamide (NAM) or nicotinamide riboside (NR) could prevent thymus atrophy and hepatosteatosis by TCDD while increasing sirtuin activity [9].

In addition to serving as a substrate for PARP and sirtuin activities [12] NAD^+^ is a cofactor for many metabolic reactions, including steps in glycolysis, the cellular energy-generating pathway that breaks down glucose to pyruvate and lactate [13]. Glycolysis is enhanced in cancer [14] and is also required in physiological conditions involving rapid cell expansion, as in developing thymocytes [15, 16]. T cell precursors from the bone marrow differentiate in the thymus into mature T-cells after passing through intermediate subpopulations named for their expression of cluster of differentiation markers, CD4 and CD8. Premature thymocytes lack CD4 and CD8 markers (double negative, DN). Later thymocytes express both markers (double positive, DP) and then they become single positive (SP), expressing either CD4 or CD8 [17]. There are 4 DN subpopulations (DN1-4) distinguished by the expression of CD44 and CD25 markers [17]. A switch from mitochondrial respiration to glycolysis is required for the transition from the DN3 to DN4 stage, when cells are highly proliferative [16]. Exposure to TCDD has been reported to decrease DN3 and DN4 populations in mouse thymus as early as 12 hours after TCDD treatment [18]. Thus we asked whether TCDD might impair glycolysis in organs targeted for its toxicity, such as the thymus.

We report here that TCDD decreases glycolysis in the thymus while increasing glycolysis in the liver. Further, boosting NAD^+^ levels normalized the effects of TCDD on glycolysis in both organs.

## Materials and methods

### Chick embryos (CE) and treatments

Fertilized eggs from White Leghorn chickens (*Gallus gallus*) (Animal Science Department, Poultry Unit, University of Connecticut) were maintained at 37°C at high humidity. Chick embryos (CE) at 15 or 16 days of development (hatching is at 21 days) were injected *in ovo* into the fluids surrounding the embryo through a hole in the shell with one or more of the following: TCDD (1 nmol per egg in 0.005 ml dioxane; a dose that produces maximal induction of 7-ethoxyresorufin deethylase (EROD), an index of TCDD mediated CYP1A4 activation [19]), dioxane (equivalent to the amount used for TCDD (solvent controls); nicotinamide riboside (NR) (Chromadex, Irvine, CA) 2 mg per egg in 0.1 ml of distilled water; nicotinamide (NAM, Sigma, St Louis MO) 10 mg per egg in 0.1 ml of distilled water; 2-deoxy-D-glucose (2-DG, Sigma, St Louis MO) 5mg in 0.05 ml of distilled water. Treatment with TCDD was for 24 hr (unless otherwise indicated) and NR or NAM was injected after administration of TCDD, 4 hr prior to dissection. Chick embryos were euthanized by rapid decapitation following Weill Cornell Medicine Institutional Animal Care and Use Committee (IACUC) guidelines.

### Lymphocyte and hepatocyte preparation

#### Lymphocytes

Lymphocytes were removed from thymus glands of CE as follows: thymus glands were squeezed between two microscope slides to release lymphocytes into a Petri dish containing 1X phosphate buffered saline (PBS) supplemented with 1% fetal bovine serum (FBS). After passing the cell solution through a 100 μm nylon mesh cell strainer, lymphocytes were pelleted by centrifugation and incubated for 5 min at room temperature with red cell lysis buffer (0.13M ammonium chloride, 0.017M Tris Base, 0.01M potassium bicarbonate) to remove red blood cells. Lymphocytes were washed twice with 1X PBS before being used to measure glucose uptake, lactate release, ATP levels or ECAR (Extracellular Acidification Rate, an index of glycolysis) as described below. *Hepatocytes*—Hepatocytes were extracted from CE liver as previously described [20], treated for 4 min with red cell lysis buffer (0.13M of ammonium chloride, 0.017M Tri Base, 0.01M potassium bicarbonate) to remove red blood cells and assayed for glucose uptake or lactate release assays as described below.

### Human primary hepatocytes

Hepatocytes from a 37 year-old female donor with liver focal nodular hyperplasia (benign tumour, no chemotherapy history) were obtained through the Liver Tissue Cell Distribution System (University of Pittsburgh, PA) and seeded in collagen coated 12-well plates (Corning) at 0.6 million cells per well in DMEM (MP#1033122) supplemented with 10% FBS, 0.01% dexamethasone (0.4 μg/ml) 0.001% glucagon (7ng/ml), 3.6% iTS mix (iTS mix was prepared by mixing 20 ml of iTS premix (Corning #354352, containing human recombinant insulin, human transferrin (12.5 mg each), selenous acid (12.5 μg), BSA (2.5 g), and linoleic acid (10.7 mg)), 30 ml of HEPES (1M, pH = 7.3) and 20 ml of Pen-Strep). After 24 hr, the human primary hepatocytes were treated for 24 hr with TCDD (10nM) or the solvent dioxane.

### Glucose uptake assay

Glucose uptake by CE lymphocytes or hepatocytes was measured using Glucose Uptake-Glo Assay kits (Promega J1342) following the manufacturer’s directions. Briefly, lymphocytes or hepatocytes, prepared as described above, were plated in 96-well plates (1 x 10^6^ cells per well) and incubated for 30 min with 2-deoxyglucose added to a final concentration of 1mM. Luminescence was measured using a SpectraMax M2e plate reader (Molecular Devices, San Jose, CA) for 1 hr, with readings taken every 2 minutes.

### Lactate release assay

Lactate release from cells was measured using the Lactate-Glo Assay kit (Promega J5021) following the manufacturer’s directions. Briefly, lymphocytes or hepatocytes, prepared as described above, were plated in 24-well plates (1–2 x 10^6^ cells per well) in 1 ml of RPMI1640 medium supplemented with 5% dialyzed FBS (Gibco, catalog#:16140063) (for lymphocytes) or Std Ham’s [20] supplemented with 2% dialyzed FBS and 25mM of glucose (for hepatocytes) and incubated overnight at 37°C. The medium was then removed from the wells, centrifuged, and 50 μl of medium was transferred to each well of a 96-well plate. The lactate detection reagent, prepared as described in the supplier’s protocol, was added to each well. Luminescence was measured using a spectrophotometer as described above.

### Extracellular Acidification Rate (ECAR)

ECAR, an index of glycolysis, was measured in lymphocytes extracted from thymus glands from CE treated *in vivo* with TCDD (0.3 million cells/well in 96 well plate), and in cultured hepatocytes (80,000 cells/well of 96 well plate) after 24 hr treatment with TCDD (1nM), using an Agilent Seahorse XF Stress glycolytic kit (Agilent Technologies, Santa Clara, CA) and a Seahorse XFe96 Analyzer, following the manufacturer’s directions. Cells were plated in RPMI 1640 (lymphocytes) or DMEM (hepatocytes) supplemented with glutamine (1mM). Oxygen Consumption Rate (OCR) was also monitored in the same conditions.

### ATP measurement

ATP was measured using ATPLite luminescence kits (PerkinElmer, Waltham, MA), following the manufacturer’s directions, in lymphocytes extracted from thymus glands. Three million cells were used per sample. Luminescence was measured using the SpectraMax M2e plate reader.

### SDS polyacrylamide gel electrophoresis (PAGE)/Western blotting

Tissue samples were homogenized in sample buffer (125 mM TRIS-HCl, 4% SDS, 16% glycerol, 10% β-mercaptoethanol, 0.002% bromophenol blue, supplemented with protease/phosphatase inhibitor (Cell Signalling, Cat. # 5872)), boiled for 5 min and centrifuged for 15 min at 14,000 rpm. Proteins were separated by electrophoresis on precast Tris-Glycine gels (Invitrogen/Life Technologies) and transferred to nitrocellulose membranes. The membranes were subjected to Western blotting with primary antibodies against AKT (Cell Signalling Technology 9272S, lot# 25), pAKT (Ser473) (Cell Signalling Technology 4060S, lot# 16), HIF1A (Aviva, 38054, lot# QC7591-41992) and beta-actin (ACTB) (Sigma A5441, lot# 116M4801V). Goat anti rabbit IgG (Sigma A6154) or mouse IgGk (Sc-516102, Santa Cruz Biotechnology, Dallas, TX) was used as the secondary antibody. Protein bands were detected using ECL Western Blotting Detection reagents (GE Healthcare, UK). Band intensities were quantified by densitometry using Syngene Software (Syngene, Frederick, MD).

### RT-qPCR

Total RNA extracted from tissues was used to prepare cDNA, as previously reported [10]. qPCR analysis was conducted using an Eppendorf Mastercycler *ep* gradient machine for 40 cycles of amplification. Accession gene numbers and primer sequences are given in *S1 File*. Fold-changes in mRNA were calculated by the standard 2^−ΔΔCt^ method [21] using 18S mRNA as a reference for normalization.

### Flow cytometry

Lymphocytes were extracted from thymus glands as described above and single cells were resuspended in 1x PBS +1% FBS (buffer) in 3 ml tubes. 0.1 μg of CD4-APC, 0.1 μg of CD8-PE and 0.5 μg CD3-FITC (SouthernBiotech, Birmingham, AL: anti-CD4-APC (cat# 8210–11, lot#: C1706-R846X); anti-CD8A-PE (cat# 8220–09; lot #: L2413Y276B); anti-CD3-FITC (cat # 8200–02, lot #: E3813-QL26B) were added to 100 μl of the lymphocyte solution containing 1 x 10^6^ lymphocytes and incubated for ½ hr in ice in the dark. Preliminary titration experiments were conducted to assess antibody requirements for optimal detection of the cell populations. After incubation, buffer was added to the top of the tubes containing the cell solutions. Cells were centrifuged, buffer was aspirated and 100 μl of fresh buffer added. Cell samples were filtered using BD Falcon Cell-Strainer Cap tubes and analysed in the Flow Cytometry Core Facility at Weill Cornell Medical College using a BD Fortessa FACS machine. Data were analysed using FlowJo software (FlowJo LLC, Becton Dickinson).

### Glyceraldehyde 3-phosphate dehydrogenase (GAPDH) activity

GAPDH activity was measured using a colorimetric GAPDH assay kit (ScienCell Research Laboratories, Carlsbad, CA) homogenizing thymus or liver (20 mg in 100 μl of lysis buffer) and following manufacturer’s directions.

### Protein measurements

Protein concentrations were measured using Biorad-DC Protein Assay (Bio-Rad Laboratories, Inc. Hercules, CA).

### Statistics

Differences between group means were evaluated using GraphPad software by unpaired, two tailed t-tests; p values ≤ 0.05 were considered statistically significant. When more than two groups were compared one-way Anova analysis was used with Tukey's honestly significant difference (HSD) test as a post hoc test. All experiments were replicated and the number of independent experiments and of CE used in each experiment are indicated in the Figure legends.

## Results

### Effects of TCDD in the thymus

#### TCDD suppresses mRNAs for glucose transporters and glycolytic genes in the thymus

**Fig 1:**
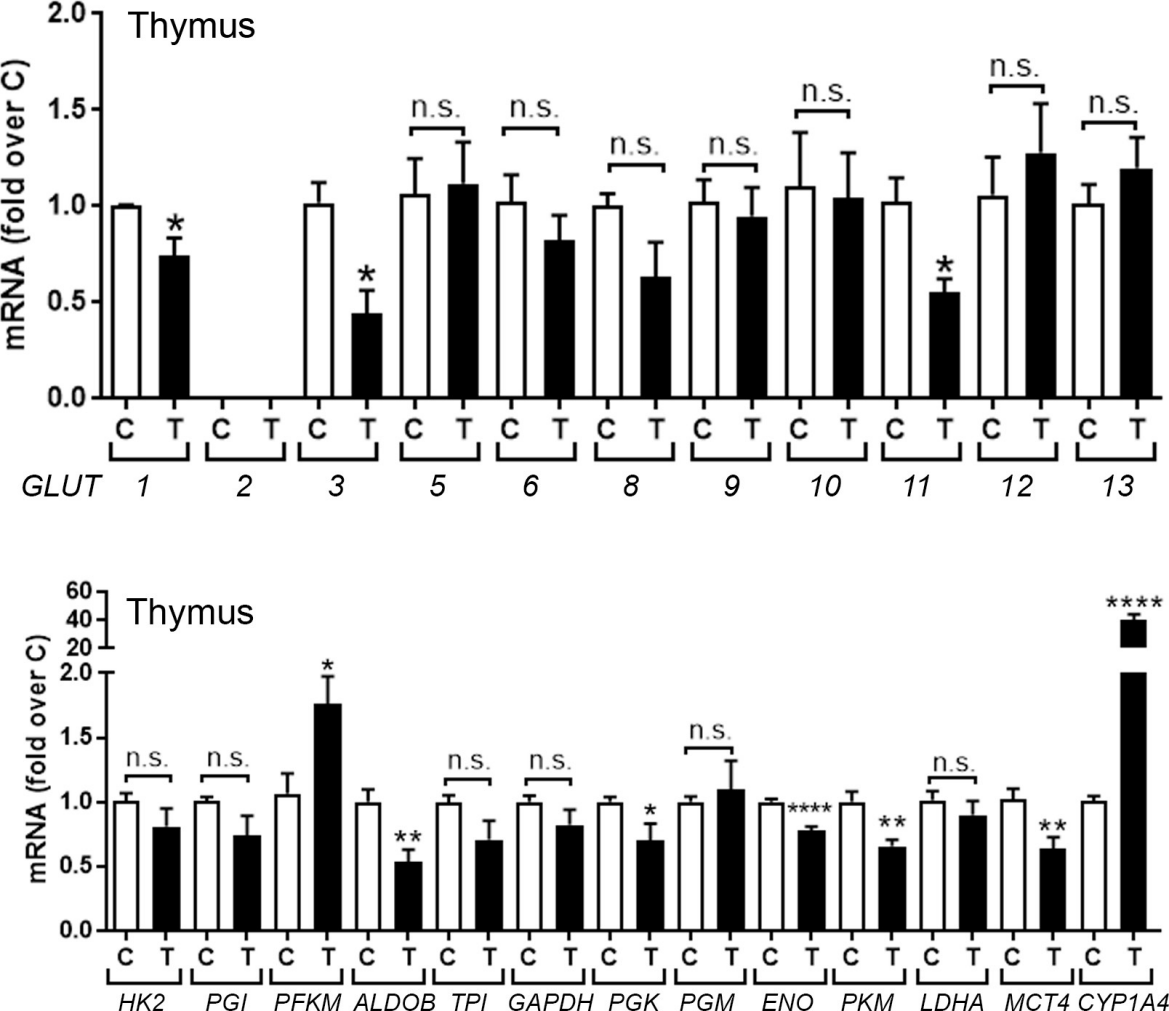
TCDD effects on mRNAs for glucose transporters and glycolytic genes in chick embryo (CE) thymus. CE were treated with TCDD (T) (1nmol/egg) or solvent dioxane (control, C). After 24 hr total RNA was extracted from thymus glands and used to perform RT-qPCRs for known chicken glucose transporters (*GLUT*) (*upper panel*) and genes involved in glycolysis (*lower panel*). *CYP1A4* mRNA was measured as an index of AHR activation by TCDD. Bar graphs show means ± SE. Differences between control (C) and TCDD-treated (T) groups was performed using t-test analysis. For this and other figures, *, p≤0.05, **; p, ≤ 0.005; ***, p ≤ 0.001; ****, p ≤ 0.0001; n.s., not significant.

**Fig 1** (*upper panel*) shows effects of treatment with TCDD or solvent for 24 hr on mRNA levels for known glucose transporters (*GLUT*, *SLC2A*) in thymus glands of chick embryos (CE). *GLUT*s *4*, *7* and *14* are not shown as they have not been identified in the chicken [22]. TCDD suppressed expression of *GLUT1*, the major glucose transporter in thymus [23, 24], along with *GLUT3* and *GLUT11*. Although chicken *GLUT2* has been identified, it was not detected in the thymus.

Parallel assessment of TCDD effects on mRNAs for genes involved in glycolysis in the thymus (**Fig 1**, *lower panel*) showed that TCDD significantly suppressed mRNAs for four glycolytic genes: aldolase (*ALDOB*), phosphoglycerate kinase (*PGK*), enolase (*ENO1*) and pyruvate kinase (*PKM*) but did not affect mRNAs for other glycolytic genes except for phosphofructokinase (*PFK)*, which was significantly increased. Lactate dehydrogenase (*LDHA*) mRNA, which converts pyruvate to lactate, was unaffected by TCDD, while TCDD significantly decreased mRNA for the monocarboxylate transporter 4, *MCT4* (*SLC16A4*), a plasma membrane transporter protein that controls the cellular efflux of lactic acid/H^+^ and is highly expressed in glycolytic cells [25]. mRNA for *CYP1A4*, a positive control to monitor AHR activation by TCDD, was increased by TCDD in the thymus. In summary, these results show that AHR activation by TCDD in the thymus mainly suppressed glucose transporters and genes associated with glycolysis.

#### TCDD suppresses indices of glycolysis and ATP levels in the thymus

**Fig 2:**
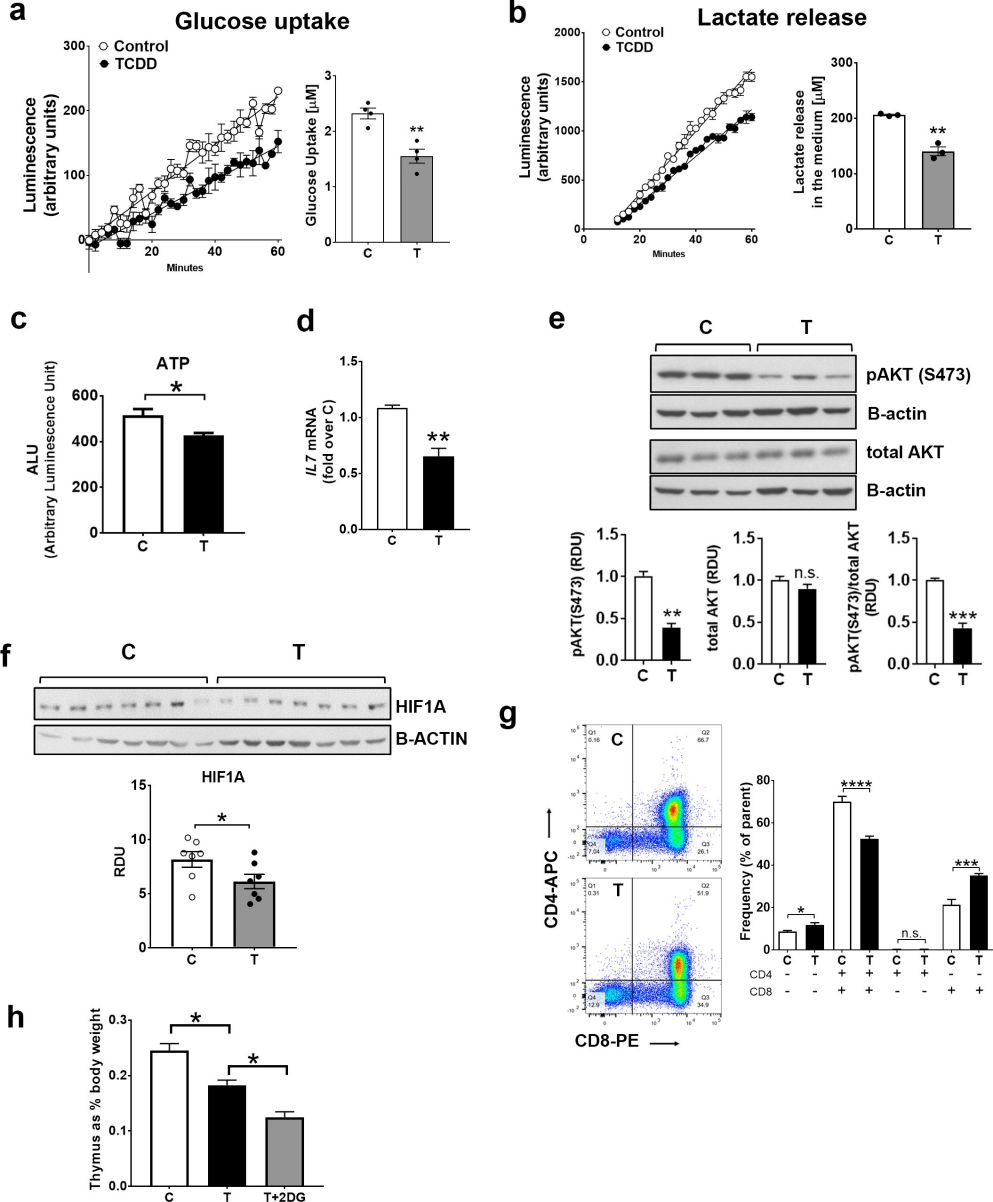
TCDD suppresses glycolytic hallmarks in thymus. CE were treated with TCDD or dioxane for 24 hr. a-b, Lymphocytes were extracted from thymus glands and used immediately to measure glucose uptake (a) or lactate release (b) as described in *Material and Methods*. Graphs for a and b show luminescence detected by a spectrophotometer over 1 hr. Bar graphs show quantification of TCDD effects on glucose uptake and lactate release. Representative experiments are shown (n = 3 for glucose uptake and n = 4 for lactate release; each experiments included 3–4 replicates for each treatment group with each replicate containing pooled thymus lymphocytes from 3–4 CE). c, ATP was measured in lymphocytes extracted from thymus glands from CE treated *in ovo* with dioxane (control) or TCDD for 24 hr (n = 2; 6–7 replicates for treatment group). d, Total RNA was extracted from thymus glands and RT-qPCR for *IL7* was performed as described in *Material and Methods*. (n = 3; 4–5 replicates for each treatment group). e-f, Thymus glands, homogenized in Laemli buffer, were used for Western blotting with pAKT (S473), total AKT, beta-actin (ACTB) and HIF1A antibodies. Representative Western blots are shown (n = 4; 3 replicates for each treatment group). g, Representative contour plots show populations of CD3^-^ thymocytes analysed for level of expression of CD4 and CD8 markers: lower left quadrant, double negative (CD4^-^CD8^-^); lower right quadrant, single positive for CD8 (CD4^-^CD8^+^); upper left quadrant, CD4 single positive (CD4^+^CD8^-^); upper right quadrant, double positive (CD4^+^CD8^+^). n = 4 independent experiments (with each experiment including 5 replicates of pooled lymphocytes from n = 4–5 CE). h, CE were treated with TCDD or dioxane for 72 hr with or without 2-DG (5mg/egg administered 3 times: at the time of the injection of TCDD and 24 hr and 48 hr after TCDD injection). For all panels in this figure, bar graphs represent means ± SE. For panels in a-g, t-test analysis comparing control (C) and TCDD-treated (T) groups was performed. For bar graph in panel h, one-way Anova analysis was used to calculate differences among the means and Tukey's honestly significant difference (HSD) test was used as a post hoc test.

To learn whether the suppression by TCDD of mRNA levels for glucose transporters and glycolytic genes in the thymus was accompanied by suppression of glycolysis, we examined effects of TCDD on two hallmarks of glycolysis [26], glucose uptake (**Fig 2A**) and lactate release (**Fig 2B**) by thymic lymphocytes assayed immediately after extraction from thymus glands of CE treated with TCDD *in ovo* for 24 hr. TCDD suppressed glucose uptake and lactate release by 27%, consistent with decreased glycolysis in the thymus. TCDD suppressed ATP levels in thymic lymphocytes by 17% (**Fig 2C**), indicating that suppression of glycolysis by TCDD in the thymus was accompanied by decreased energy production.

**Fig 2D and 2E** show that in thymus glands TCDD treatment for 24 hr decreased two factors known to stimulate thymus growth: IL7 and phosphorylated AKT (pAKT, at serine 473) [16, 27, 28], consistent with roles for these factors in decreased glycolysis by TCDD in the thymus.

HIF1A (hypoxia inducible factor 1 alpha), like phosphorylated AKT, promotes glycolysis by enhancing expression of glycolytic genes [29]. The low oxygen tension in the thymus favours HIF1A stabilization [30, 31]. TCDD decreased HIF1A levels in thymus (**Fig 2F**), an effect that would be expected to contribute to decreased glycolysis. Our evidence that TCDD decreased several factors involved in glycolysis in the thymus, *IL7* mRNA, pAKT and HIF1A, supports roles for these factors in decreased glycolysis in the thymus by TCDD.

To learn if the suppression of glycolytic endpoints by TCDD was accompanied by effects on thymocyte precursors, we conducted flow cytometry experiments using lymphocytes extracted from thymus glands of CE treated for 24 hr with TCDD or dioxane. Average changes from four flow cytometry experiments (**Fig 2G)** showed that TCDD increased the double negative CD4^-^CD8^-^ population by 26% while decreasing the CD4^+^CD8^+^ double positive population by 24%. TCDD increased the CD8^+^ single positive population (by 45%). These results show that decreased glycolysis by TCDD in the thymus is accompanied by a decrease of CD4^+^CD8^+^ double positive cells, the precursors of mature CD4^+^ and CD8^+^ single positive lymphocytes.

To assess the role of suppressed glycolysis in thymus atrophy by TCDD we treated CE with TCDD with or without the glycolysis inhibitor 2-deoxy-D-glucose (2-DG). CE were treated for 72 hr with TCDD (we previously showed this treatment duration lowered thymus weight by TCDD [9]) with and without 2-DG. **Fig 2H** shows that cotreatment with TCDD+2DG exacerbated TCDD effects on thymus atrophy supporting a role for suppressed glycolysis in TCDD effects in the thymus.

#### NAD^+^ boosting corrects TCDD effects on glycolysis and ATP levels in thymic lymphocytes

**Fig 3:**
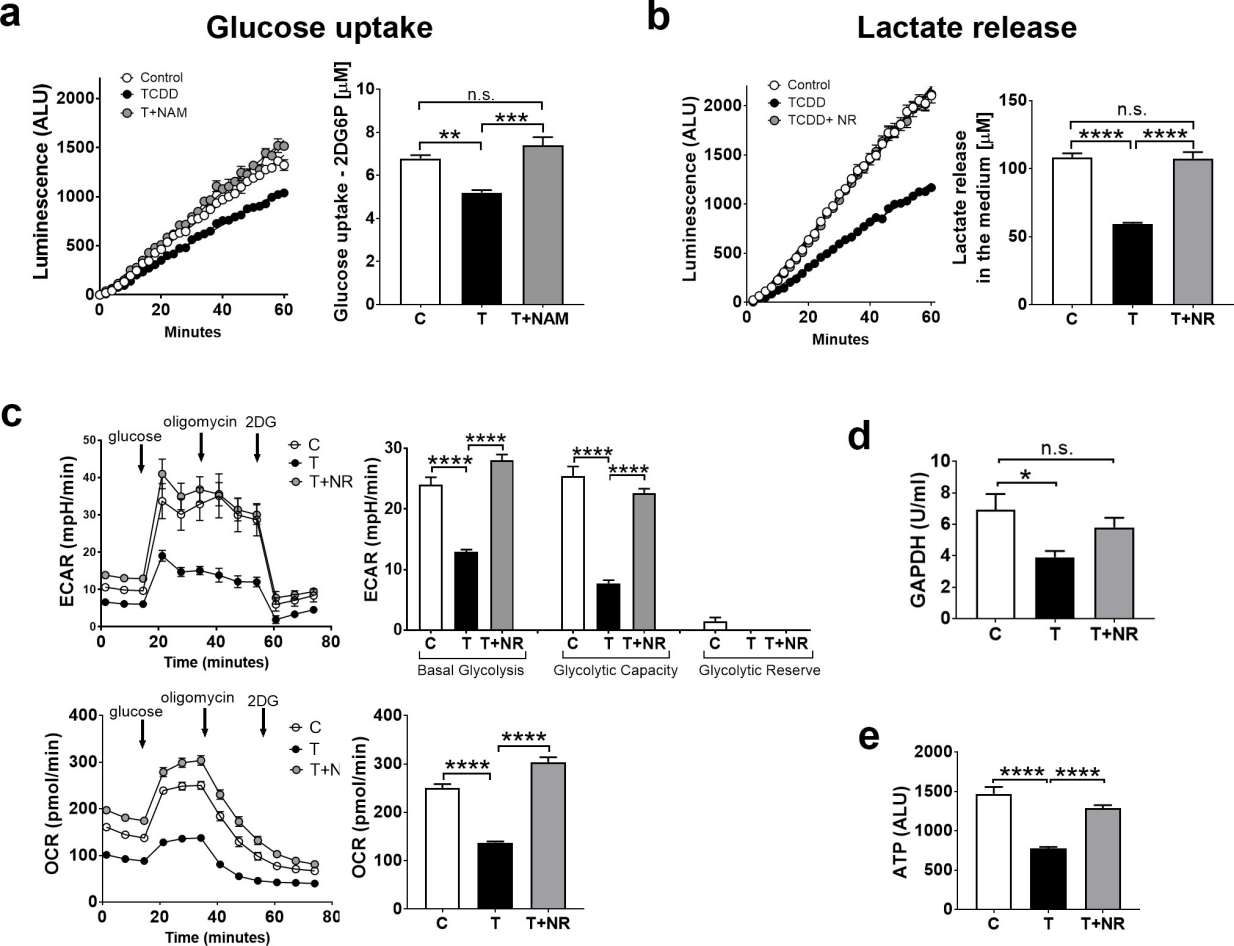
Correction of TCDD effects on glycolytic endpoints in CE thymus by the NAD^+^ precursors nicotinamide (NAM) or nicotinamide riboside (NR). CE were treated with TCDD (T) or solvent (C) for 24 hr. NAM (10mg) or NR (2mg) were administered for the last 4 hr before the chick embryos were sacrificed. Assays for glucose uptake (a) and lactate release (b) by lymphocytes are described in *Material and Methods*. c. Lymphocytes extracted from thymus glands of CE were used to measure the Extracellular Acidification Rate (ECAR, glycolytic index) by using an Agilent Seahorse XF Stress glycolytic kit. Oxygen Consumption rate (OCR) was measured in the same conditions. *Bar graphs*, quantification of ECAR and OCR results. d. GAPDH activity was measured using thymus gland lysates as described in *Material and Methods* (n = 4 or 5 replicates for treatment group). e. ATP measured in lymphocytes extracted from thymus glands of CE treated as above (6 replicates of thymus lymphocytes from a pool of 4–5 CE for each treatment group). For all the panels in this figure, bar graphs represent means ± SE; one-way Anova analysis was used to calculate differences among the means and Tukey's honestly significant difference (HSD) test was used as a post hoc test.

We previously reported that treatment of chick embryos with NAD^+^ precursors, nicotinamide (NAM) or nicotinamide riboside (NR), increased NAD^+^ levels in both thymus and liver and could prevent thymus atrophy and hepatosteatosis by TCDD [9]. We show here that NAD^+^ boosting also ameliorated TCDD effects on glycolysis in thymic lymphocytes. Thus, NAM or NR at doses that increased NAD^+^ levels in CE thymus glands [9], given 4 hr before sacrificing CE treated with TCDD 24 hour earlier, increased glucose uptake (**Fig 3A**) and lactate release (**Fig 3B**) by thymic lymphocytes. NR did not affect lactate release by lymphocytes in the absence of TCDD in these conditions (**S1 Fig in S1 File**, *left bar graph*). Further, we used Seahorse Technology to measure the extracellular acidification rate (ECAR, an index of glycolysis in living cells [26]). We found that TCDD decreased basal ECAR by about 50% and glycolytic capacity by 70% in lymphocytes extracted from thymus glands of CE treated with TCDD *in ovo* (**Fig 3C**, *upper panels*), consistent with our other evidence that TCDD decreased glycolysis in the thymus. Administration of NR for the last 4 hr of TCDD treatment restored ECAR to control levels, confirming that NAD^+^ boosting ameliorated the effects of TCDD on glycolysis in thymic lymphocytes. TCDD decreased also oxygen consumption rate in the conditions in which glycolysis was assayed and NR treatment abrogated this effect (**Fig 3C**, *lower panels*). We then measured effects of TCDD with and without NR on the activity of glyceraldehyde 3-phosphate dehydrogenase (GAPDH), a glycolytic enzyme that requires NAD^+^ as a substrate. We found that TCDD decreased GAPDH activity in the thymus and NR treatment corrected this effect (**Fig 3D**). Further, NR normalized ATP levels by TCDD in the thymus, thereby correcting the energy defect caused by TCDD in the thymus (**Fig 3E**).

### Effects of TCDD in the liver

#### TCDD increases mRNAs for glucose transporters and glycolytic genes in CE liver and in human primary hepatocytes

**Fig 4:**
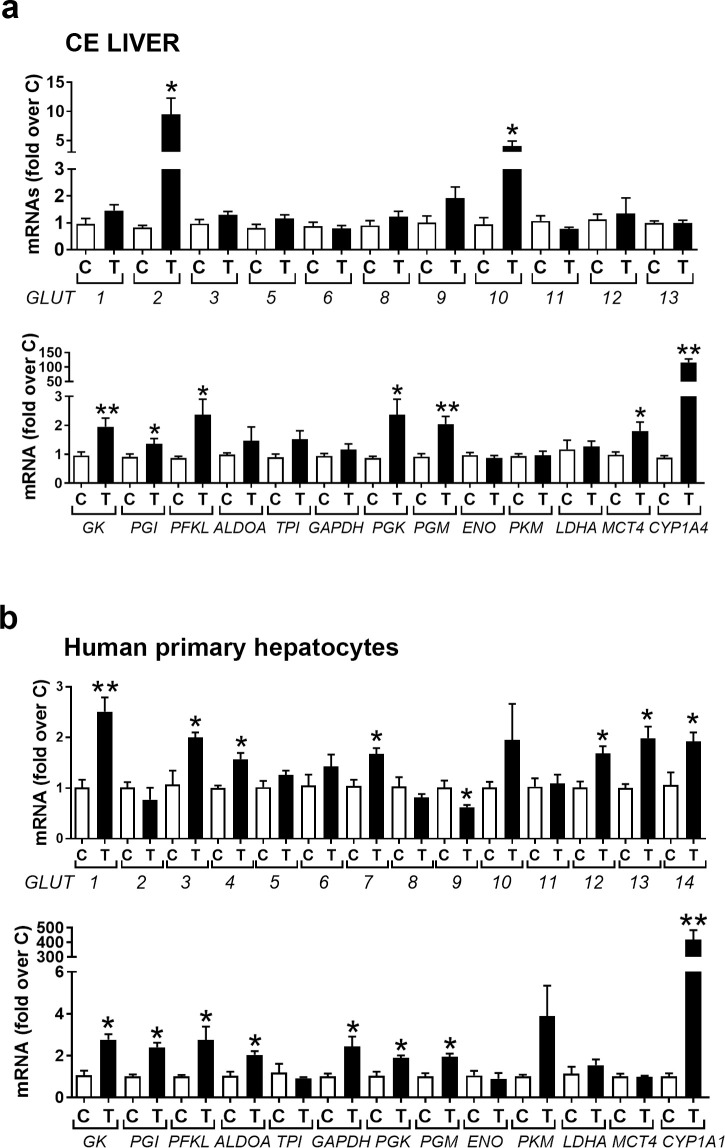
TCDD effects on mRNAs for glucose transporters and glycolytic genes in CE liver and human primary hepatocytes. a. CE were treated with TCDD or dioxane and after 24 hr livers were removed and total RNA was extracted and used to perform RT-qPCRs for chicken *GLUT*s (*upper panel*) and glycolytic enzymes (*lower panel*). b. Human primary hepatocytes were plated in a 12-well plate (0.6 million cells/well). After 24 hr they were treated with TCDD (10 nM) for a further 24 hr before being collected for total RNA extraction and analysis by RT-qPCR for human *GLUT*s (*upper panel*) and enzymes involved in glycolysis (*lower panel*). n = 3 replicates for each treatment group. Bar graphs show means ± SE. Differences between control (C) and TCDD-treated (T) groups was performed using t-test analysis.

In liver, in contrast to the thymus, after 24 hr treatment TCDD increased mRNAs for *GLUT2* and *GLUT10* (**Fig 4A**, *upper panel*) and for some glycolytic genes including the hepatic specific hexokinase (glucokinase, *GK*), phosphoglucoisomerase (*PGI*), phosphofructokinase, liver type (*PFKL*), phosphoglycerokinase (*PGK*), phosphoglyceromutase (*PGM*) and *MCT4*, which transports lactate out of the cell (**Fig 4A**, *lower panel*). In human primary hepatocytes TCDD treatment for 24 hr increased mRNAs for *GLUT1*, *3*, *4*, *7 12*, *13*, *14* (**Fig 4B**, *upper panel*) (while decreasing mRNA for *GLUT9*). TCDD also increased mRNAs for glycolytic genes except for *ENO*, *LDHA* and *MCT4* (**Fig 4B**, *lower panel*), which were unaffected by TCDD. Thus, in contrast to the thymus, TCDD mainly increased mRNAs for genes associated with glycolysis in CE liver and human primary hepatocytes.

#### TCDD increases glycolytic indices in CE liver; NAD^+^ boosting diminishes those TCDD effects

**Fig 5:**
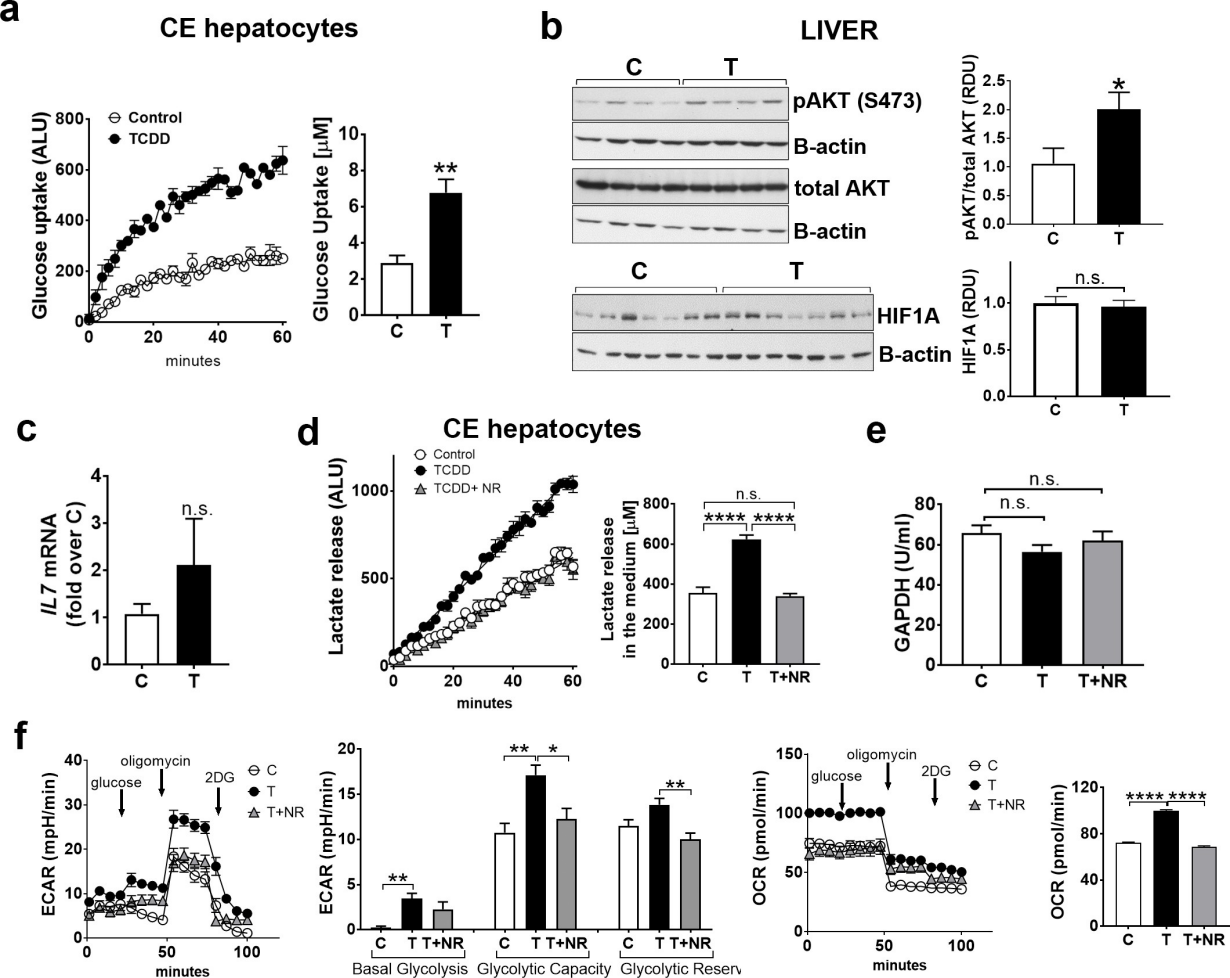
TCDD increases glycolytic hallmarks in the liver; NAD^+^ boosting normalizes glycolysis. a. CE were treated with TCDD or solvent dioxane and after 24 hr livers were removed and hepatocytes were extracted and assayed for glucose uptake (a). Graph shows luminescence detected by a spectrophotometer over 1 hr period. Bar graph shows quantification of the effect of TCDD on glucose uptake. A representative experiment is shown (n = 4 replicates for each treatment group). b, *Upper panels*, Livers from CE treated with TCDD (24hr) or solvent (C) were homogenized in Laemli buffer and used for Western blotting with the indicated antibodies. Representative Western blots are shown (n = 3 independent experiments with 3 replicates for each treatment group in each experiment). The bar graph shows relative densitometry units for bands for pAKT over total AKT. *Lower panels*, Homogenates of livers from CE treated for 24 hr with TCDD or solvent were used for Western blotting with antibodies for HIF1A and beta-actin (30 μg of total protein/lane). c, Total RNA was extracted from livers of CE treated as above and RT-qPCR for *IL7* was performed as described in *Material and Methods*. (n = 4; 3–5 replicates for each treatment group). d, Lactate release from hepatocytes extracted from livers of CE treated with TCDD for 24 hr with and without NR added for the last 4 hr of the TCDD treatment. Graph shows luminescence detected by a spectrophotometer over 1 hr. Bar graph shows quantification of effects of TCDD with or without NR on lactate release. A representative experiment is shown (n = 3–4 replicates for each treatment group; each treatment group contained pooled lymphocytes from 4–5 CE). e, GAPDH activity was measured as described in *Material and Methods* using lysates of livers from CE treated as described in d (n = 4 or 5 replicates for treatment group). f, CE hepatocytes were cultured and treated for 24 hr with TCDD (1nM). Extracellular Acidification Rate (ECAR) was measured by Seahorse technology as described in *Material and Methods*. Oxygen Consumption rate (OCR) was measured in the same conditions as ECAR. For all panels, bar graphs represent means ± SE. For a-c, differences between control (C) and TCDD-treated (T) groups was performed using t-test analysis. For d-f, one-way Anova analysis was used to calculate differences among the means and Tukey's honestly significant difference (HSD) test was used as a post hoc test.

Also, in contrast to thymus, TCDD treatment for 24 hr increased glucose uptake by hepatocytes extracted from livers of CE compared to the controls (**Fig 5A**) and increased pAKT (S473) (**Fig 5B**, *upper panels*). TCDD did not affect HIF1A levels in liver homogenates (**Fig 5B**, *lower panels*). TCDD effects on *IL7* mRNA in the liver were variable and there was no significant difference between control and TCDD treated liver samples (**Fig 5C**).

TCDD increased lactate release by hepatocytes from CE treated with TCDD for 24 hr compared to control (**Fig 5D**). Cotreatment with TCDD and NR for the last 4 hr corrected lactate release by hepatocytes to control levels (**Fig 5D**). NR did not affect lactate release by CE hepatocytes in the absence of TCDD (**S1 Fig in S1 File**, *right bar graph*). GAPDH activity, which was decreased in the thymus by TCDD, was not significantly affected by TCDD in the liver nor was it affected by NR (**Fig 5E**). Seahorse analysis conducted in cultured CE hepatocytes showed that TCDD increased basal glycolysis and maximum glycolytic capacity (**Fig 5F**) and that TCDD and NR cotreatment decreased glycolytic capacity and reserve. Thus increasing NAD^+^ levels by NR corrected TCDD effects on hepatocyte glycolysis. Oxygen consumption rate (OCR), which was monitored in the conditions in which glycolysis was assessed, was also increased by TCDD and normalized to control levels by cotreatment with TCDD+NR. We then study the effects of the glycolysis inhibitor 2DG on triglyceride (TRG) content in the liver. CE were treated with TCDD (72 hr) with and without 2-DG as described above for thymus. Cotreatment with TCDD+2DG did not prevent increased TRG content in the liver (*data not shown*), suggesting that increased glycolysis alone is not responsible for increased TRG by TCDD.

## Discussion

The major new findings reported here are: 1) AHR activation by TCDD disrupts glycolysis; 2) TCDD has different organ-specific effects on glycolysis in two organs that are sites of TCDD toxicity, decreasing glycolysis in the thymus and increasing it in the liver. Thus, TCDD decreased mRNAs for glucose transporters and glycolytic enzymes in the thymus in association with decreased glucose uptake and lactate release by thymic lymphocytes. In contrast, TCDD increased mRNAs for glucose transporters and glycolytic genes in the liver, effects accompanied by increased glucose uptake and lactate release by hepatocytes; 3) NAD^+^ boosting using NAD^+^ precursors (which we previously showed could prevent TCDD toxicities in the thymus and the liver [9]) normalized glycolysis in both organs; 4) we found further that effects of TCDD on glycolytic genes pertained also in human primary hepatocytes, demonstrating that the findings in CE extend beyond avian species.

In the thymus TCDD decreases levels of three factors known to stimulate glycolysis: *IL7*, pAKT and HIF1A. The decrease in these factors, alone or together, could contribute to the suppression of glycolysis by TCDD in thymus. Thus, IL7 produced by thymic epithelial cells (TEC) [32] binds to the IL7 receptor on the lymphocyte surface, activating AKT [28], which in turn increases expression of glycolytic genes [14, 33] and has been shown to be essential for thymocyte development and survival [27]. Further *IL7* KO mice exhibit thymus atrophy [34] such as occurs with TCDD treatment. Interestingly, impairment of thymocyte development and thymus atrophy in “normal” aging also has been reported to be accompanied by decreased IL-7 production [35, 36].

Thus suppression of IL7 expression by TCDD could contribute to the decrease of pAKT levels by TCDD in the thymus. AKT signalling is an important factor in thymocyte development [16, 37]. Hence, the decrease of pAKT by TCDD in the thymus could also contribute to changes in subpopulations of developing thymocytes after TCDD treatment (**Fig 2G**) as well as to decreased glycolysis by TCDD. The changes in thymus subpopulations by TCDD shown here are consistent with findings of others for TCDD effects on thymic subpopulations in mouse models [18, 38, 39].

Future studies should investigate the role of suppressed HIF1A in the effects of TCDD on glycolysis in the thymus and how TCDD decreases HIF1A. Interestingly, Hening Lin’s group [40] showed that TiPARP targets HIF1A for ADP-ribosylation increasing HIF1A degradation suggesting the novel possibility that TiPARP induction by TCDD might play a role in decreasing HIF1A in the thymus.

Data from the Seahorse experiments showed that addition of glucose to thymic lymphocytes increased ECAR (i.e. glycolysis) (**Fig 3C**). ECAR was not further increased by the addition of oligomycin, an inhibitor of mitochondrial respiration, suggesting that glycolysis is working at full capacity in the lymphocytes, and supporting the importance of this energy pathway in developing lymphocytes. In agreement with these findings, thymocytes removed from thymus glands have been reported to consume 95% of added glucose by glycolysis [41], underlining the importance of glycolysis in thymus metabolism.

Oxygen consumption rate, an index of mitochondrial respiration, was also decreased by TCDD in lymphocytes (and increased by TCDD in hepatocytes) in the conditions in which we measured ECAR. Treatment with NR was able to normalized OCR in both lymphocytes and hepatocytes. Future studies are needed to understand whether the effects of TCDD on mitochondrial respiration are a consequence of those on glycolysis or whether TCDD has independent effects on mitochondrial respiration.

The evidence that TCDD has opposite effects on glycolysis in liver and thymus is also noteworthy. A recent paper by Han *et al*. [42] reports that TCDD increases pAKT in mouse liver cells, consistent with our findings. There is other evidence for TCDD having different effects in different organs: for example, TCDD affected expression of different genes in testes and liver [43], TCDD decreased PEPCK activity in liver while increasing it in kidney and brown adipose tissue in a rat model [44] and thymus specific effects of the AHR activation have been attributed to cross talk with pathways of other transcriptional factors including HIF1A [45].

It is possible that increased glycolysis by TCDD in the liver could reflect decreased SIRT6 activity by TCDD in this organ. Thus, we previously showed that TCDD decreased levels and activity of hepatic SIRT6 in CE liver (see Fig 4 and S10 Fig in [9]) and loss of SIRT6 has been associated with increased transcription of glycolytic genes also in a mouse model [46–48]. Further studies are needed to understand whether effects on sirtuins by TCDD participate to TCDD effects in glycolysis.

qPCR results (**Fig 4**) showed that TCDD affected mRNAs for different members of the GLUT family in chick embryo liver and human primary hepatocytes. Discrepancies could be due to species differences (for example chicken lack GLUT 4, 7 and 14 and characterization of all the chicken GLUTs is not fully understood [22]) as well as *in vivo* and *in vitro* conditions used for CE and human primary hepatocyte experiments, respectively.

Treatment with the glycolysis inhibitor 2-DG exacerbated effects on thymus atrophy by TCDD, supporting a role for decreased glycolysis in TCDD effects in the thymus. However, treatment with 2-DG did not ameliorate hepatosteatosis by TCDD. Future studies are needed to understand the role of increased glycolysis by TCDD in the liver.

The ability of NAD^+^ boosting to correct effects of TCDD on glycolysis in both thymus and liver also merits comment. Although NAD^+^ boosting *in vivo* has been reported to be beneficial and corrective in different pathologies and tissues, the metabolic changes leading to the corrective effects of NAD^+^ boosting are not well understood [49, 50]. As seen here in the thymus, administration of NAD^+^ precursors has been shown to increase glycolysis in contexts where glycolysis was suppressed [51, 52] and corrected the decrease of GAPDH activity by TCDD. But NAD^+^ is also a substrate of sirtuins [11] and increasing sirtuin activity by NAD^+^ boosting would be expected to decrease glycolysis [49], as we see in the liver. Further studies will be required to understand how NAD^+^ boosting affects glycolysis in different organs and pathophysiological contexts.

Finally, as tissue NAD^+^ levels decline with aging [53] as with TCDD [9], decline of NAD^+^ could contribute to age-related effects of thymus involution and hepatic dysfunction of glucose and energy metabolism in the aging population, thus NAD^+^ boosting might also be a useful strategy against age-related pathologies.

## Supporting information

S1 Raw images (PDF)

S1 File (PDF)

## Abbreviations

AHR: aryl hydrocarbon receptor
CE: chick embryo
GLUT: glucose transporter
TCDD: 2,3,7,8-tetrachlorodibenzo-*p*-dioxin
