# Supplementary material for: Organ-specific effects on glycolysis by the dioxin-activated aryl hydrocarbon receptor
Source: PLoS One. 2020 Dec 15;15(12):e0243842. doi: 10.1371/journal.pone.0243842 (PMC7737989; doi:10.1371/journal.pone.0243842)

Original Western blots included in **Figure 2e**

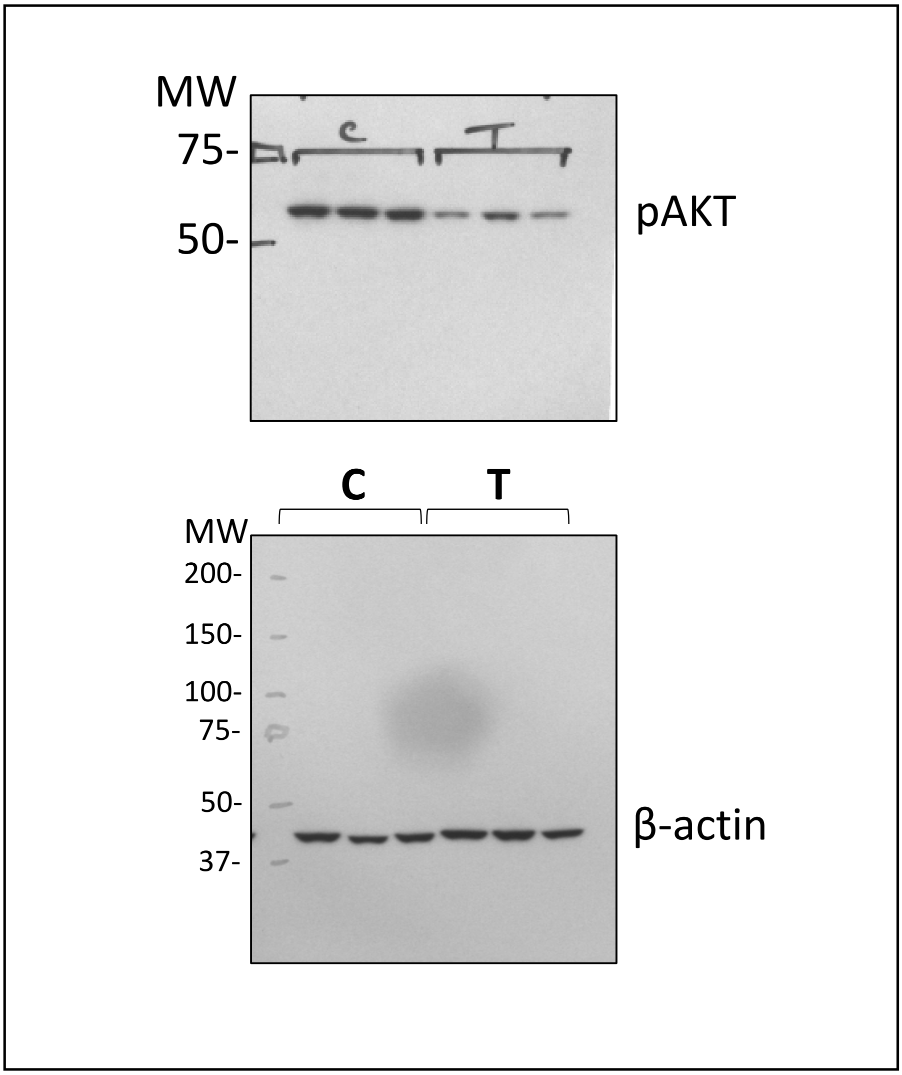

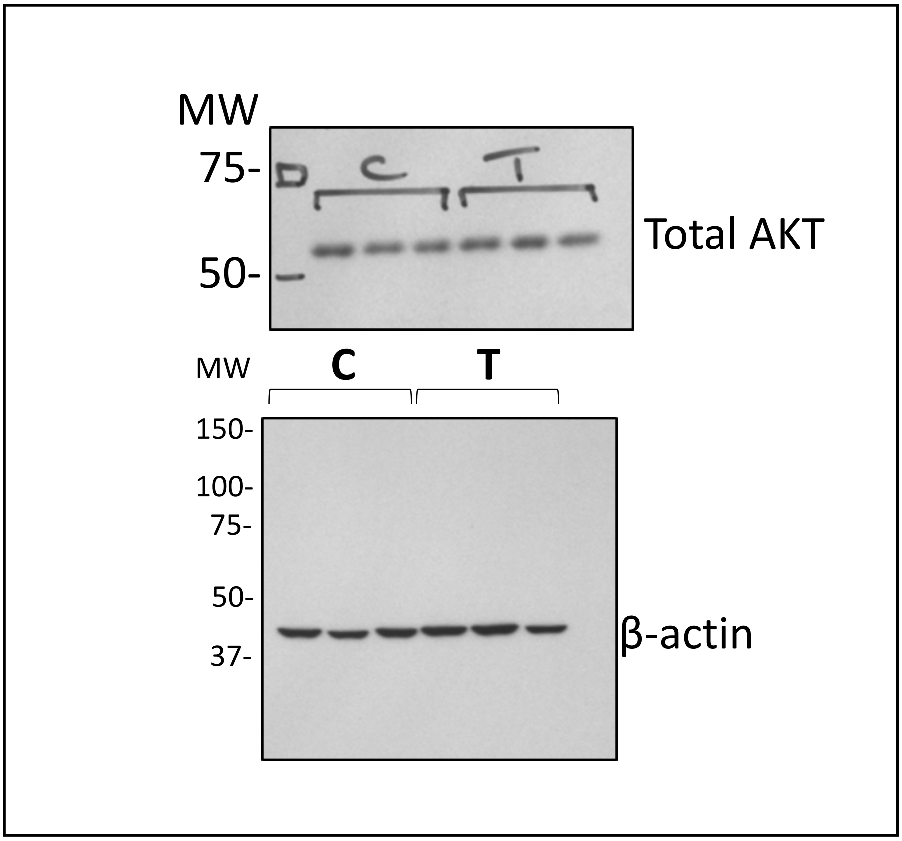

Original Western blots included in **Figure 2F**

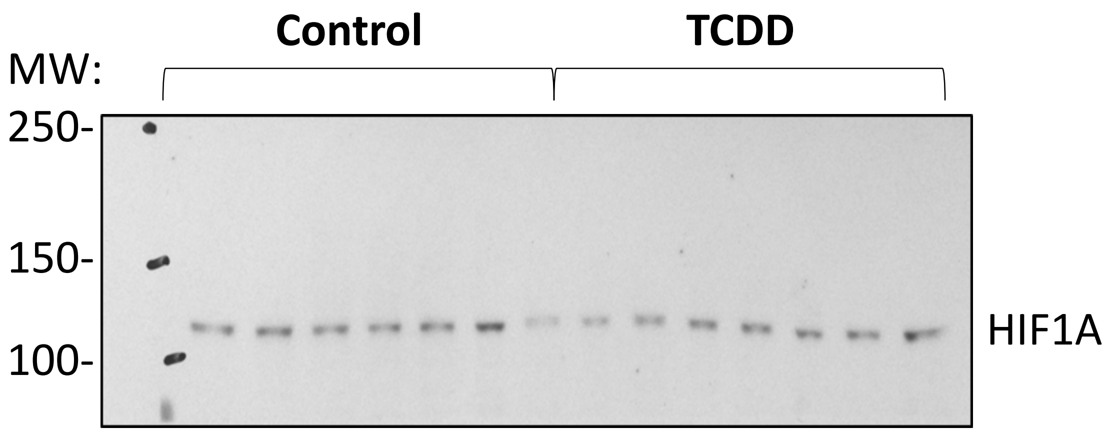

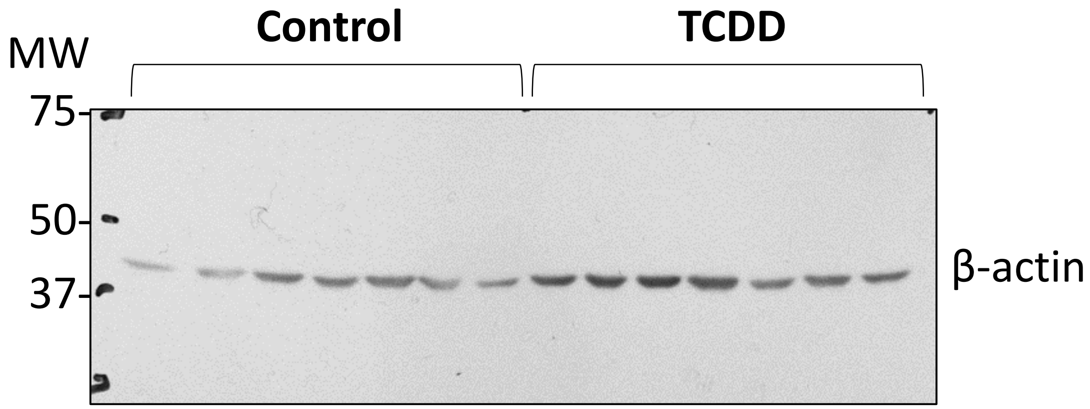

Original Western blots included in **Figure 5b**

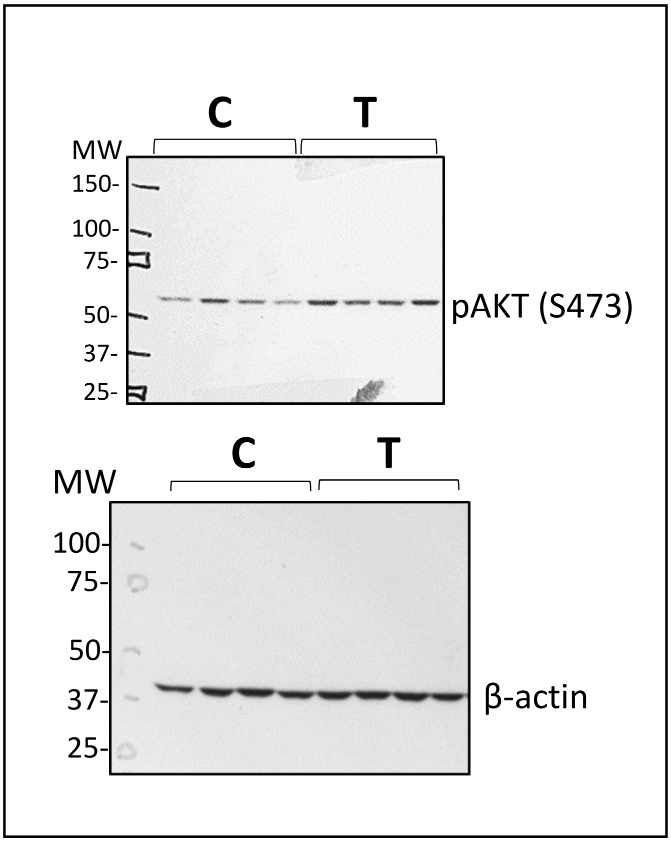

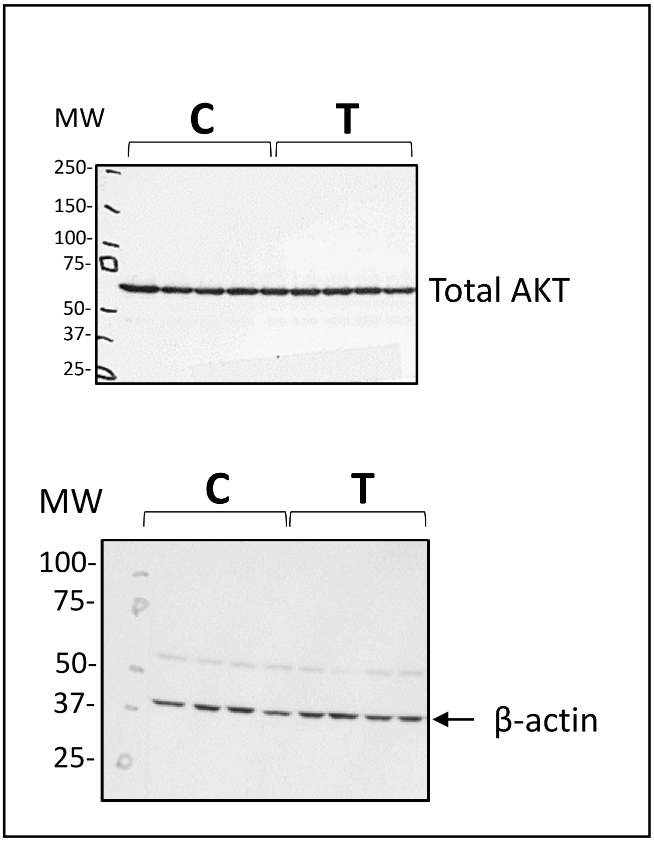

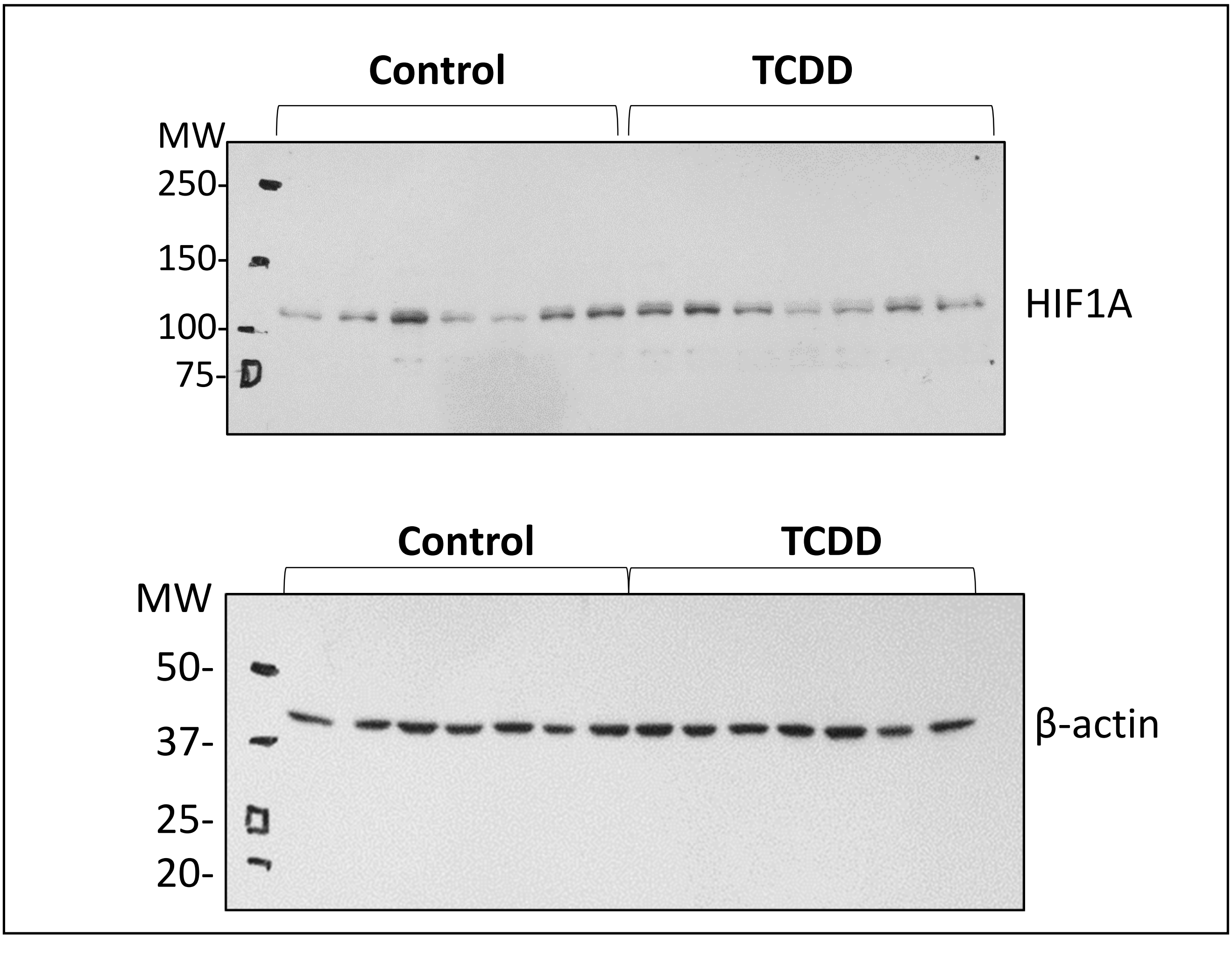

Supplement: S1 Raw images — (PDF) [file pone.0243842.s001.pdf]
