# Supplementary material for: Organ-specific effects on glycolysis by the dioxin-activated aryl hydrocarbon receptor
Source: PLoS One. 2020 Dec 15;15(12):e0243842. doi: 10.1371/journal.pone.0243842 (PMC7737989; doi:10.1371/journal.pone.0243842)
Supplement: S1 File — (PDF) [file pone.0243842.s002.pdf]

Supporting Information

Chicken primers used for qPCRs:

Gene	Accession #	Forward	Reverse
GLUT1	NM_205209.1	atatgaggagcccatcagcc	aggaaggcgaggatgtttga
GLUT2	NM_207178.1	ggtggtcaatgtcctctcca	cactcacatacatgggcacg
GLUT3	NM_205511.1	tccaggccttctacaacagg	gaagcgcccaattatcagca
GLUT5	XM_025142667.1	ccattgttgctgcagtcctt	aggatgccaagggtgatgaa
GLUT6	XM_025141409.1	tggggctctacatccacttg	agggtgatgtagttggtggg
GLUT8	NM_204375.1	acaccgagatttctgctgga	tgcctgttacttgctggaga
GLULT9	XM_420789.6	agctagggtgatgtagggga	tggcactccttcccattcat
GLUT10	XM_417383.6	tcaactgctgcccatactga	aacaggtcgggtaaggagtg
GLUT11	NM_001347709.1	tgctacgtcccattccttgt	ctctctctgtgtccctgcaa
GLUT12	XM_419733.5	gcacacacaggctacacaaa	gaacctgagacccttgagct
GLUT13	XM_001232939.5	aagggctctgtttcctcctc	caaaacttcctcccagtgcc
ALDOB	NM_001007977.3	gtgaccatgatctccaacgc	gattccaggaacagcagcag
HK2	NM_204212.1	cttggagagcacgtgtgatg	catgatggccgagaagtgtg
PGI	NM_001006128.1	ttcactgagaaccgagctgt	tcagagccaccaatcccaat
PFK	XM_015278795.2	actcatcaagggaagtggca	cccacagcacattctcatcg
TPI	NM_205451.1	tattggagctgcatgggtga	taccagttccgatagcccaaa
GAPDH	NM_204305.1	gggtcttatgaccactgtcc	gtaagcttcccattcagctcag
PGK2	NM_204985.2	ctccaaaggaaccaaagccc	tccagtgctaacgtggctaa
PGM1	NM_001031556.2	aaaccctgacccagctctac	agtaggggaggctacagtga
ENO1	NM_205120.1	ggcgtatctctggctgtttg	acgttggttgcatcctttcc
PKM	XM_015278796.2	actcatcaagggaagtggca	cccacagcacattctcatcg
LDHα	NM_205284.1	agctgaaaggagaaatgctt	tgggaatgatgaatttgaag
MCT4	NM_204663.1	acgctgtgtctacctcttca	aaacttctgagtgccaacaa
IL7	NM_001037833.1	ttctccttgttctgtcgcca	gctcttcgatgtcatggctg
CYP1A4	NM_205147.1	ggacggaggctgacaaggtg	tgctgcaggatggtggtgag
18S	AF173612.1	gaccataaacgatgccgact	agacaaatcgctccaccaac

Human primers used for qPCRs:

**Gene**	**Accession #**	**Forward**	**Reverse**
GLUT1	NM_006516.3	tggcatcaacgctgtcttct	ctagcgcgatggtcatgagt
GLUT2	NM_000340.2	cagggcgacgttctctcttt	ggcctggcccaatttcaaag
GLUT3	NM_006931.3	cactgttccctctgctcctg	cacatgattctcccgcctca
GLUT4	NM_001042.3	ctggatgccattgctcatgc	agagtgcccctatcatgggt
GLUT5	NM_001135585.2	tagcaggccatggacaaagg	gctgtcattgctatgccacg
GLUT6	NM_017585.4	gagcacaggaagagcagtgt	agggttgtatgcactcctgc
GLUT7	NM_207420.2	gggaccctgctgatcaacaa	caccactcgggaaaagacga
GLUT8	NM_014580.5	tcgtgaccaaggagttcagc	agtcacaggcttgctccatc
GLULT9	NM_020041.3	aaggagatccgtggctctct	tcagccaggacctcctctac
GLUT10	NM_030777.4	ccttggtctgctggtgtcat	caagcaggtttgttggcctc
GLUT11	NM_030807.5	actcagggagctcctaggtg	ggcttgtcacctgtctcctc
GLUT12	NM_145176.3	ctgtccttagccagcttgct	catggctcgtcctctgatcc
GLUT13	NM_052885.4	tacaaatgaggcagcctggg	aggcattggtcccattccag
GLUT14	NM_001286233.2	tttctcatcccacgcactcc	cggccaatccctcctgaaat
HK2	NM_000189.5	atggagaaagggcttggagc	ccaaagcacacggaagttgg
PGI	NM_000175.5	gccaaggatccttctgcagt	ctcccacccaatcccagaac
PFKL	NM_002626.6	gtcttcgactgcaggaccaa	catgcggtgctcgaaatcag
ALDOA	NM_184041.4	cagggacaaatggcgagact	gatctcaggctccacgatgg
TPI	NM_000365.6	ggactcggagtaatcgcctg	gagacgttggacttcagcca
GAPDH	NM_002046.7	gagaaggctggggctcattt	ggcatggactgtggtcatga
PGK	NM_000291.4	gcccatgcctgacaagtact	ctccacatgaaagcggaggt
PGM	NM_002629.4	cagaagagagcgatccggac	ggtgggacatcataggagcg
ENO1	NM_001428.5	ttcggtcacctgttggctac	ggtggaaagtgaggcgagaa
PKM	NM_002654.6	ggaagtgggcagcaagatct	gctcgaccccaaacttcaga
LDHA	NM_005566.4	accgtgttattggaagcggt	tagcccaggatgtgtagcct
MCT4	NM_030777.4	gctgggccacaagtattcct	gtcagtcccatcccagaacg
CYP1A1	NM_000499.5	aaacagggccacatagatgc	agggtcctggtttggctagt
18S	X03205.1	ggccgttcttagttggtgga	cccggacatctaagggcatc

Figure S1

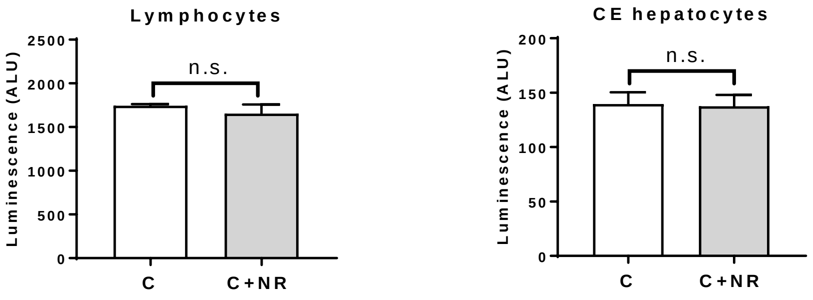

Figure S1 – Nicotinamide riboside (NR) did not affect lactate release by control CE lymphocytes and hepatocytes. Thymic lymphocytes (*left bar graph*) or hepatocytes (*right bar graph*) were assayed immediately after extraction from thymus glands and livers, respectively, of CE treated with nicotinamide riboside for 4 hr. Bar graphs show means ± SE. Differences between control (C) and TCDD-treated (T) groups was performed using t-test analysis. n.s., not significant.
